# Influenza Virus Ribonucleoprotein Complexes Gain Preferential Access to Cellular Export Machinery through Chromatin Targeting

**DOI:** 10.1371/journal.ppat.1002187

**Published:** 2011-09-01

**Authors:** Geoffrey P. Chase, Marie-Anne Rameix-Welti, Aurelija Zvirbliene, Gintautas Zvirblis, Veronika Götz, Thorsten Wolff, Nadia Naffakh, Martin Schwemmle

**Affiliations:** 1 Department of Virology, University of Freiburg, Freiburg, Germany; 2 Spemann Graduate School of Biology and Medicine (SGBM), University of Freiburg, Freiburg, Germany; 3 Institut Pasteur, Paris, France; 4 Institute of Biotechnology, Vilnius University, Vilnius, Lithuania; 5 Robert Koch-Institute, Berlin, Germany; Johns Hopkins University - Bloomberg School of Public Health, United States of America

## Abstract

In contrast to most RNA viruses, influenza viruses replicate their genome in the nucleus of infected cells. As a result, newly-synthesized vRNA genomes, in the form of viral ribonucleoprotein complexes (vRNPs), must be exported to the cytoplasm for productive infection. To characterize the composition of vRNP export complexes and their interplay with the nucleus of infected cells, we affinity-purified tagged vRNPs from biochemically fractionated infected nuclei. After treatment of infected cells with leptomycin B, a potent inhibitor of Crm1-mediated export, we isolated vRNP export complexes which, unexpectedly, were tethered to the host-cell chromatin with very high affinity. At late time points of infection, the cellular export receptor Crm1 also accumulated at the same regions of the chromatin as vRNPs, which led to a decrease in the export of other nuclear Crm1 substrates from the nucleus. Interestingly, chromatin targeting of vRNP export complexes brought them into association with Rcc1, the Ran guanine exchange factor responsible for generating RanGTP and driving Crm1-dependent nuclear export. Thus, influenza viruses gain preferential access to newly-generated host cell export machinery by targeting vRNP export complexes at the sites of Ran regeneration.

## Author Summary

Influenza viruses replicate their single-stranded RNA genomes in the nucleus of infected cells. Since new virus particles are formed at the plasma membrane, these genomes must be exported in the form of a viral ribonucleoprotein complex (vRNP) from the nucleus to the cytoplasm at a late point during infection. We have discovered that this nuclear export process involves an intermediate step whereby the vRNPs are very tightly tethered to a specific region of dense chromatin. Although the tight tethering of a complex which should be very mobile seems paradoxical, we found that this close association between vRNPs and host cell chromatin brought the viral complexes into close proximity with Rcc1, a protein involved in regenerating the host cell export machinery. Through this targeting, the virus gains access to the recycled host cell export proteins before they are able to find a cellular substrate. Thus, the virus hijacks a vital host process not by direct competition, but by finding a location from which to “snatch” the host protein complexes immediately after generation.

## Introduction

Influenza viruses are nearly unique among RNA viruses, in that they perform all of their viral RNA synthesis in the nucleus of infected cells. While this exceptional attribute provides some advantages to the virus, such as access to capped cellular pre-mRNAs and the host splicing machinery, it also presents the challenge of importing and exporting the viral genome during early and late infection, respectively. Like other negative-strand RNA viruses, the influenza virus genome is encapsidated by the nucleoprotein NP, and is associated with the trimeric viral polymerase complex consisting of the PA, PB1, and PB2 proteins. This complex, known as the viral ribonucleoprotein complex (vRNP), is the minimal infectious unit that is exported from the nucleus at late time points of infection.

The nuclear export of influenza A vRNPs has been well-studied, yet many details remain unclear. First reports implicated both the viral matrix protein M1 as well as the viral nuclear export protein NEP as crucial co-factors [1]–[3]; however, the requirement for each of these proteins has subsequently been questioned [4], [5]. vRNP export was shown to be dependent on the cellular export receptor Crm1, and accordingly cytoplasmic accumulation of vRNPs can be blocked by leptomycin B [5], [6], a potent inhibitor of Crm1 [7]. However, both NP and NEP bind Crm1 and can be exported [2], [5], [8], and thus it is unclear which protein actually drives vRNP export. The current “daisy-chain” model of vRNP nuclear export postulates that M1 binds directly to vRNPs, while NEP acts as a bridge between M1 and Crm1 to facilitate translocation [9]. Despite evidence of binary interactions between each of these components, a fully-formed vRNP export complex has not been isolated from infected cells.

Crm1-dependent nuclear export is driven by a gradient of RanGTP:RanGDP between the nucleus and the cytoplasm. Crm1 association with its export cargo occurs cooperatively in a multi-protein complex containing RanGTP and other factors [10], [11], [12]. This Crm1-RanGTP-cargo complex is escorted through the nuclear pore complex to the cytoplasm [13], where RanGTP is hydrolyzed and the cargo complex dissociates [14]. After nuclear re-import of RanGDP, further export cycles require the regeneration of RanGTP, which is facilitated by the Ran guanine exchange factor Rcc1, a process chromatin-bound during the exchange process [11]. Complex formation of Ran, Crm1 and Rcc1 was shown in biochemical experiments to be facilitated by the chaperone protein RanBP3; however, this has not been confirmed in living cells [12].

Our goal in this work was to investigate both the composition of influenza vRNP export complexes and their interplay with the host cell nucleus by taking advantage of our previously-established affinity purification of vRNPs from infected cells [15]. Using a subnuclear fractionation, we identified putative vRNP export complexes which, surprisingly, were tightly bound to the nuclear substructure. Interestingly, targeting of vRNPs to these chromatin-bound nuclear domains retained Crm1 at these same domains, and thus allowed the virus to usurp the cellular export machinery. The mechanism of this usurpation appears to occur via “snatching” of recycled Crm1-RanGTP complexes after their nucleotide exchange by Rcc1 on chromatin. Thus, influenza virus gains preferential access to host cell export machinery by locating vRNPs proximal to the sites of RanGTP regeneration.

## Results

### vRNPs accumulate on chromatin upon nuclear export inhibition

**Figure 1:**
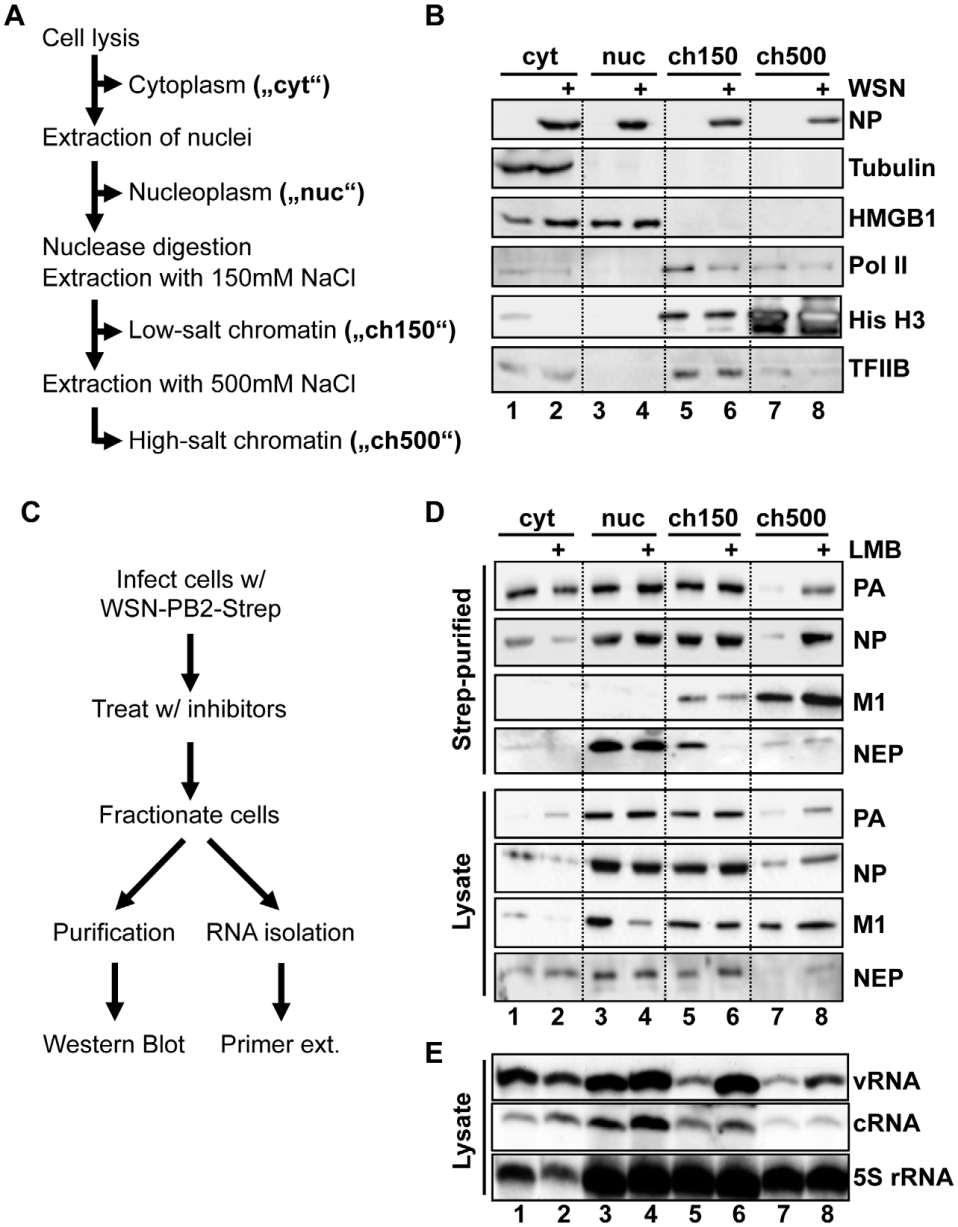
Purification of vRNPs after subcellular fractionation. (A) Schematic diagram of subcellular fractionation as described in Materials and Methods. (B) 1×10^9^ HeLa cells were infected with WSN at an MOI of 3 for 9 h or mock-infected. Cells were fractionated as described in (A), and equal amounts of protein from each fraction were analyzed by Western blot analysis for NP or cellular marker proteins. (C) Schematic workflow of Strep purification after LMB treatment. (D) 1×10^9^ HeLa cells were infected with rWSN-Strep at an MOI of 3 for 9 h. At 3hpi, cells were treated with 10 nM LMB or 0.2% EtOH vehicle. Fractionation and Strep-purification were performed as described in (C), and lysates and eluates were analyzed by Western blot for indicated proteins. (E) Cells were infected, treated, and fractionated as in (D), except that 50 U/ml DNase I was used as a nuclease, and total RNA was extracted from each fraction. RNA was analyzed by primer extension using probes specific for segment 6 v/cRNA and 5S rRNA.

To identify the components of a vRNP export complex, we performed vRNP purifications from infected cells at a late time point after infection. Based on previous reports that vRNPs within the nucleus can be either soluble or associated with chromatin [16]–[19], we postulated that subnuclear fractionation before purification might segregate vRNPs by their respective functions and thus better facilitate purification of homogenous vRNP export complexes. We therefore separated cells into cytoplasmic (“cyt”), soluble nucleoplasmic (“nuc”), 150 mM NaCl-extractable chromatin (“ch150”), and 500 mM NaCl-extractable chromatin (“ch500”) fractions (Fig. 1A). Prior to salt extraction, chromatin was digested with a nuclease. Successful fractionation was confirmed by Western blot detection of subcellular marker proteins such as tubulin (cytoplasm), HMGB1 (cytoplasm and nucleoplasm), Pol II or histone H3 (chromatin) (Fig. 1B). In agreement with previous reports [20], [21], differential salt extraction separated chromatin into fractions containing higher or lower levels of markers of active cellular transcription (Pol II, TFIIP, Fig. 1B lanes 5–8), indicative of an approximate functional distinction between the ch150 and ch500 fractions. Interestingly, we repeatedly observed a depletion of Pol II in the ch150 fraction of infected cells, which likely represents the well-described degradation of Pol II during infection. [22], [23]

To obtain a high yield of intact vRNPs from infected cells, we used a recombinant WSN strain encoding a C-terminally Strep-tagged PB2 (rWSN-PB2-Strep). The recombinant virus was generated as previously described [15], [24], and grew to similar titers as an untagged recombinant WSN (rWSN, Fig. S1A). Performing Strep-purification using lysates from rWSN-PB2-Strep-infected cells yields a specific purification of vRNPs with high efficiency (Fig. S1B+C). By infecting cells with rWSN-PB2-Strep followed by fractionation and Strep-purification, we were able to obtain high yields of vRNPs from all 4 fractions assayed (Figs. S1D + E). To differentiate between soluble trimeric polymerase complexes and fully-formed vRNPs, we assayed by Western blot the amounts of co-purified PA, as a marker for the trimeric polymerase complex, as well as co-purified NP as a marker for vRNA/cRNA-containing vRNPs. Silver staining of purified vRNPs from all fractions confirmed that PA and PB2-Strep were always purified in equimolar amounts (data not shown), indicating that PB2 is always integrated into a polymerase complex in our protocol (monomeric, purified PB2-Strep was not detected, data not shown). Despite digestion of DNA and RNA using Benzonase prior to chromatin extraction, the ratio of co-purified NP was not reduced (Fig. S1F), suggesting that the vRNP structure remains intact during this fractionation procedure. Thus, Strep-purification after subnuclear fractionation yielded biochemically equivalent vRNPs from all fractions examined.

In order to enrich vRNP export complexes in the nucleus, we performed the fractionation/purification experiments in HeLa cells with or without the Crm1 inhibitor leptomycin B (LMB), which can potently inhibit the export of influenza vRNPs [7](Fig. 1C). Analysis of vRNP accumulation by Western blot showed an expected decrease of fully-formed vRNPs in the cytoplasm after LMB treatment (Fig. 1D, lanes 1–2, “NP”). Surprisingly, a corresponding increase in nuclear vRNPs after LMB treatment was only found in the chromatin fraction extracted with 500 mM NaCl (Fig. 1D lanes 7–8 “ch500”). The vRNPs derived from this fraction also contained M1, a necessary vRNP export co-factor. Small amounts of NEP were also associated with these complexes, which were enhanced after LMB treatment. Interestingly, NEP also associated with nucleoplasm-derived vRNPs; however, these vRNPs contained no detectable M1 and did not accumulate in the nucleus after LMB treatment, suggesting a different role of NEP in these complexes (see Discussion). These experiments were also performed in A549 cells, yielding similar results (data not shown).

To verify that the complexes enriched in the ch500 fraction after LMB treatment represent vRNP export complexes and not vRNP replication intermediates, we analyzed the amounts of vRNA and cRNA in each fraction using primer extension analysis. vRNA was slightly depleted in the cytoplasm and enriched in both the ch150 and ch500 fractions after LMB treatment (Fig. 1E), while no significant differences in cRNA accumulation were observed. Thus, vRNA, but not cRNA, accumulates on chromatin after treatment with LMB. We observed discrepancies between PA/NP accumulation in Fig. 1D and vRNA accumulation in Fig. 1E, which is likely due to the need to use DNase I, instead of Benzonase, as the nuclease in the latter experiment, which leads to a altered chromatin extraction profile (data not shown). However, in both conditions vRNPs and their components associated with chromatin when vRNP export was inhibited.

**Figure 2:**
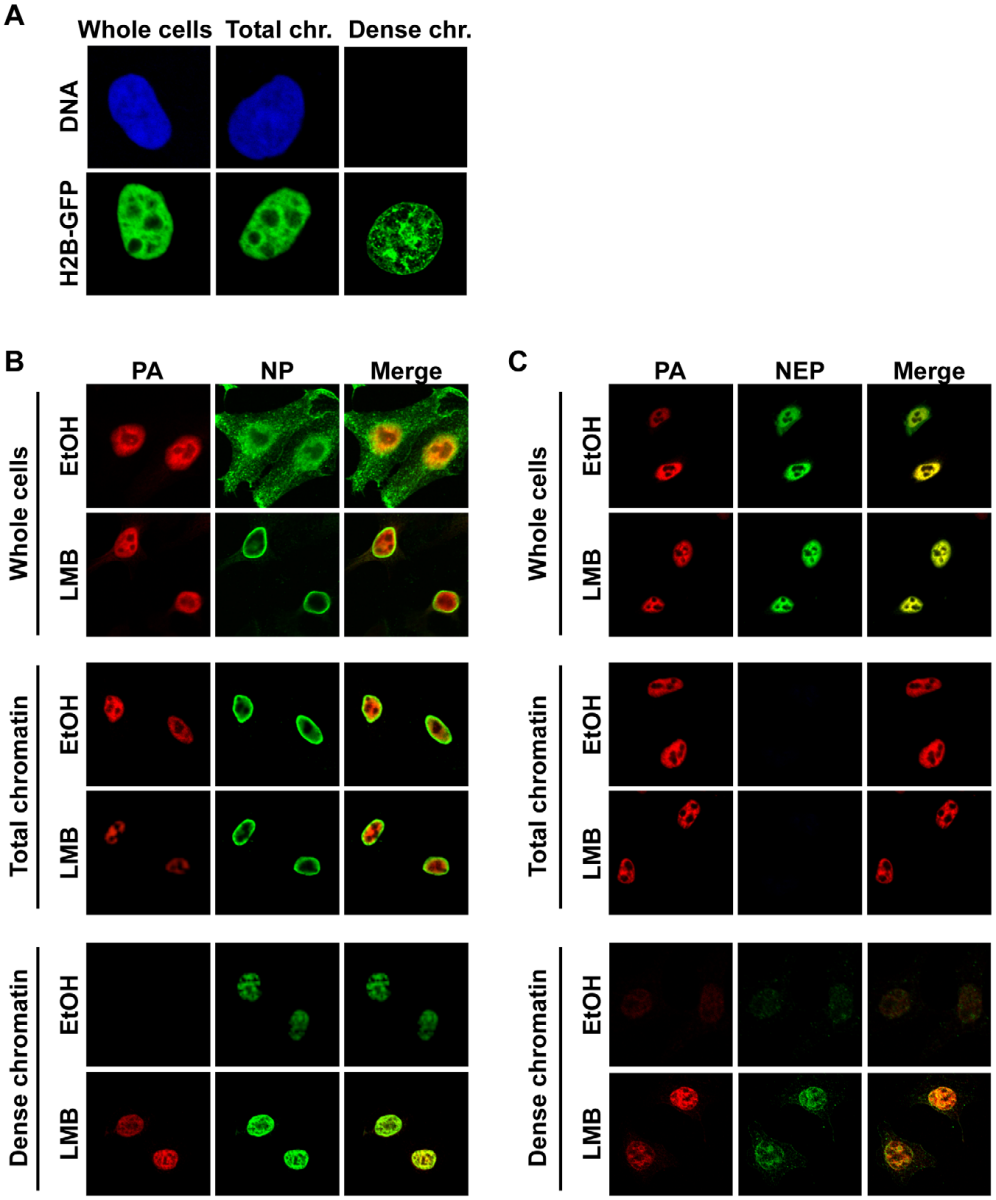
Association of vRNP export complexes with subchromatin structure. (A) The fractionation protocol was adapted to cells grown on coverslips. Cells were fixed after different fractionation steps to reveal the localization of unextracted proteins. HeLa cells were transfected with histone H2B-GFP or stained with ToPro3 after fixation to demonstrate extraction efficiency. Whole cells  =  no extraction, Total chr.  =  detergent extraction (all soluble proteins removed), Dense chr.  =  low-salt-extractable chromatin removed. (B) HeLa cells were grown on coverslips, infected with WSN at an MOI of 3 for 9 h, and treated with 10 nM LMB or 0.2% EtOH at 3 hpi. Cells were fractionated on the coverslips, fixed after fractionated, and stained for immunofluorescence using antibodies against PA and NP. Optical sections were obtained by confocal microscopy, and detector settings remained constant for each protein between all samples. (C) As in (B), except using antibodies against PA and NEP.

Since nuclear export is presumed to be a very dynamic process [25], we were surprised that vRNP export complexes were tightly associated with host cell chromatin and sought a confirmation of this finding using a separate method. We postulated that, if vRNP export complexes are indeed formed on less-soluble chromatin, the major components of these complexes should colocalize in an immunofluorescence analysis (IFA) in cells from which soluble proteins and salt-extractable chromatin has been removed, and the presence of these proteins should be enriched after LMB treatment. To test this hypothesis, we adapted a previously-described in situ extraction for influenza virus-infected cells on coverslips [17]. In a first step, all soluble proteins from the cytoplasm and nucleus were extracted, leaving chromatin-bound material intact (“Total chromatin”, Fig. 2A). This chromatin was then digested with DNase I, and extracted with 250 mM NaCl to reveal the less-soluble dense chromatin (which we so named due to the observation that histones associated with dense packing are not soluble at this concentration [26]), and its associated proteins (Fig. 2A, “Dense chromatin”). We used this extraction process to analyze the localizations of PA, NP, and NEP by IFA in infected cells with or without LMB treatment. In unfractionated cells, NP was retained in the nucleus upon LMB treatment as expected (Fig. 2B, “Whole cells”), whereas when soluble proteins were removed to reveal all chromatin-bound proteins, we observed prominent NP nuclear rings as previously described (Fig. 2B, “Total chromatin”, [27], [28]). However, we detected no difference in staining intensity between LMB-treated and untreated cells, and surprisingly, little colocalization between NP and PA. In contrast, after extraction to reveal dense chromatin-bound material, a strong colocalization of NP and PA was seen in a reticular pattern (Fig. 2B, “Dense chromatin”), which also overlapped with the localization of histone H2B (Fig. S2). This appearance of antigens previously not detectable was surprising, but is a well-known phenomenon likely caused by the exposure of epitopes previously covered by other proteins [29]. As expected, an increase in staining intensity of both PA and NP was observed after LMB treatment on the dense chromatin, but not on total chromatin or whole cells (Fig. 2B).

Staining of fractionated cells for NEP revealed a similar distribution as PA in unfractionated cells (Fig. 2C, “Whole cells”), but no NEP was detected after soluble proteins were removed (Fig. 2C, “Total chromatin”). When dense chromatin was exposed, no NEP was detectable in control cells, but a faint staining was observed in LMB-treated cells, which colocalized with PA (Fig. 2C, “Dense chromatin”). Similar PA, NP, and NEP distributions after fractionation were observed when MDCK or A549 cells were infected, or when HeLa cells were infected with influenza A strain SC35M (data not shown). Taken together, these results suggest that colocalization of vRNP export complex components can only be visualized when both soluble proteins and soluble chromatin-bound material is removed from the cell. Furthermore, after LMB treatment, vRNP protein components are enriched only on this dense chromatin fraction.

**Figure 3:**
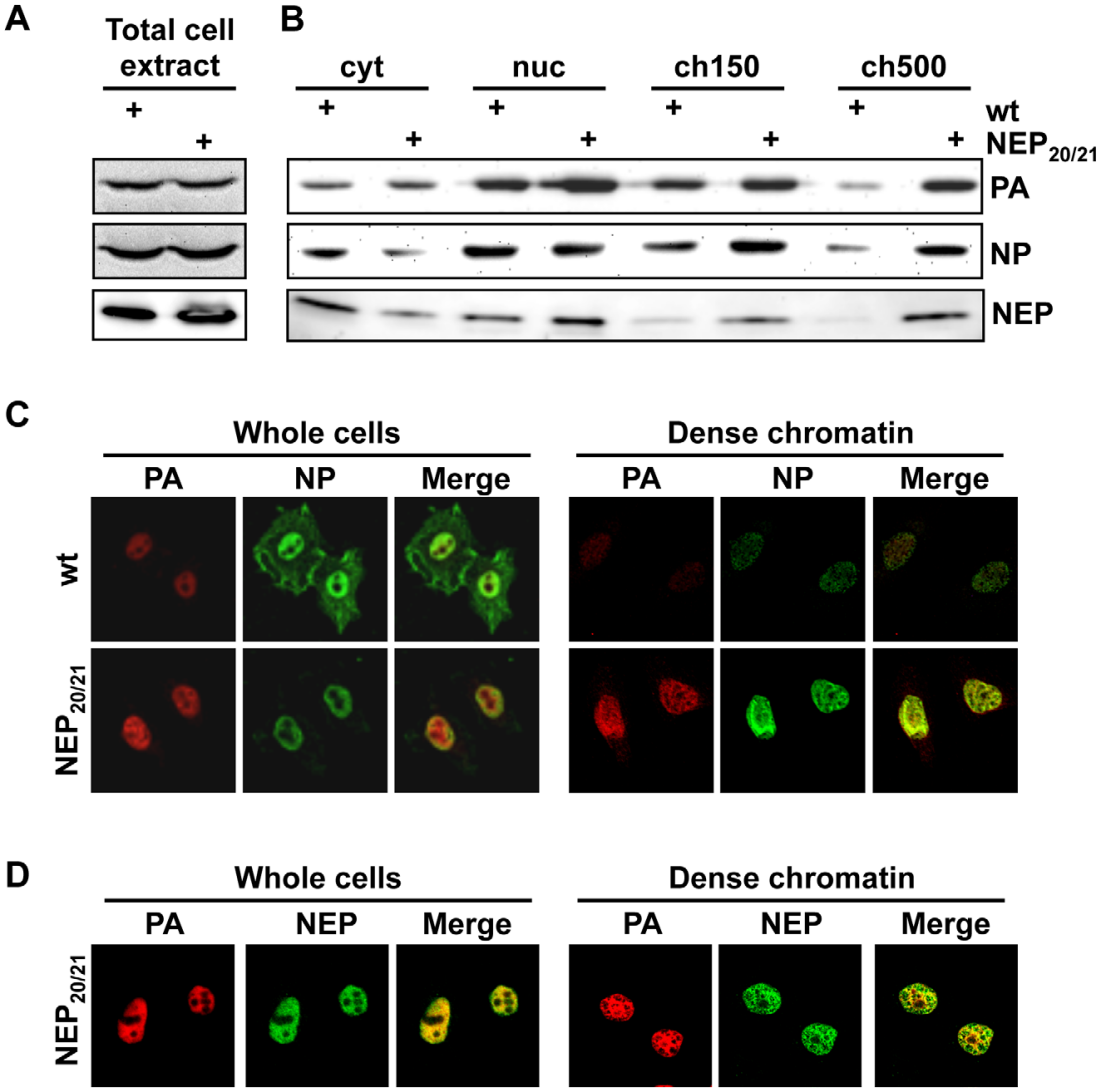
Accumulation of vRNPs on chromatin during infection with WSN-NEP_20/21_. (A) HeLa cells were infected with WSN or WSN-NEP_20/21_ at an MOI of 3 for 7 h. Whole-cell lysates were analyzed by Western blot for accumulation of PA or NP. (B) 1×10^9^ HeLa cells were infected with WSN or WSN-NEP_20/21_ at an MOI of 3 for 9 h, then fractionated as in Fig. 1B. Fractionated lysates were analyzed by Western blot for PA, NP or NEP accumulation. (C) HeLa cells on coverslips were infected with WSN or WSN-NEP_20/21_ at an MOI of 3 for 9 h, then fractionated as described in Fig. 2B and stained for IFA using antibodies against PA and NP. (D) HeLa cells on coverslips were infected with WSN-NEP_20/21_ at an MOI of 3 for 9 h, then fractionated as described in Fig. 2A and stained for IFA using antibodies against PA and NEP.

Since LMB targets the general cellular export receptor Crm1, treatment of infected cells may have unspecific effects on vRNP distribution in the nucleus. We therefore generated a recombinant WSN virus (WSN-NEP_20/21_) containing two previously-described mutations (Q20L, L21C) [30] in the nuclear export sequence (NES) of NEP. This mutant virus can synthesize vRNPs at the same rate as wt (Fig. 3A) but has a delayed vRNP nuclear export [30] (Fig. 3C, left panels). Fractionation of WSN-NEP_20/21_-infected cells at 10 hpi revealed an accumulation of PA, NP and NEP in the ch500 fraction compared to wt WSN (Fig. 3B). Correspondingly, colocalization and enrichment of PA, NP and NEP on the dense chromatin in WSN-NEP_20/21_-infected cells were observed by IFA (Fig. 3C and 3D, right panels). Thus, vRNPs accumulate in this fraction during infection when export is impaired through a suboptimal NES.

**Figure 4:**
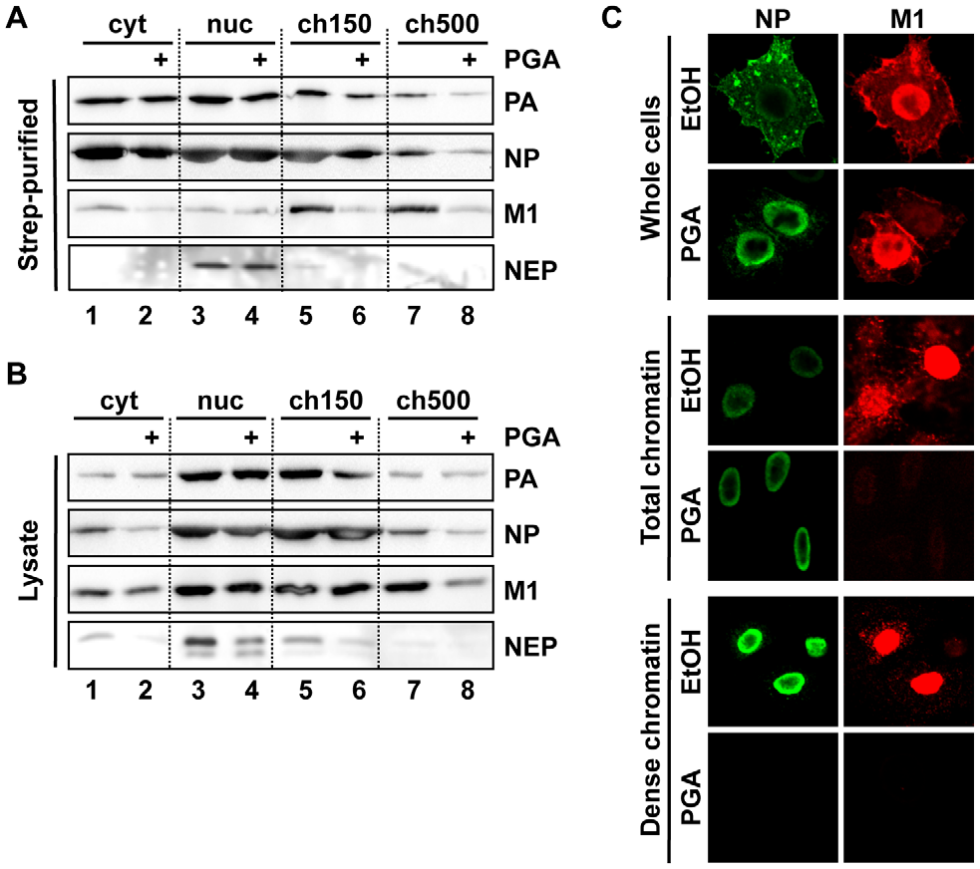
Depletion of chromatin-bound vRNPs after PGA treatment. (A) Cells were infected and purified as in Fig. 3A, except that treatment was performed using 20 µg/ml PGA at 3 hpi. Eluted complexes were analyzed by Western blot. (B) Lysates from (A) were analyzed by Western blot. (C) HeLa cells grown on coverslips were infected as in Fig. 4B, treated at 3 hpi with 20 µg/ml PGA, fractionated at 9 hpi as in Fig. 2A, and analyzed by immunofluorescence using antibodies specific for NP and M1. Note that the detector sensitivity was increased for the dense chromatin panels to demonstrate the absence of NP and M1 after PGA treatment.

To confirm the importance of high-affinity chromatin association for vRNP export, we purified vRNPs from fractionated cells after treatment with a second chemical inhibitor with a different mechanism. Among the previously-described inhibitors of influenza vRNP nuclear export, prostaglandin A (PGA) was shown to block export by inducing overexpression of Hsp70, which in turn prevents the association of M1 with vRNPs and therefore the assembly of vRNP export complexes [31]. When we performed fractionation/purification experiments after PGA treatment, we observed a decrease in vRNPs in the ch500 fraction (Figs. 4A and B, lanes 7+8). This decrease corresponded to a lack of detectable NP or PA on the dense chromatin layer by IFA after PGA treatment (Fig. 4C, lower panels and data not shown). Despite the decrease in vRNPs in the ch500 fraction, no decrease in the total amounts of PA or NP was observed in PGA-treated cells (Fig. S3), as previously reported. Concomitantly, a large decrease in the amount of chromatin-bound M1 was detected in PGA-treated cells both by Western blot (Fig. 4B) and IFA (Fig. 4C). These results suggest that Hsp70 overexpression prevents the association of vRNP-M1 complexes with chromatin, thereby leading to a lack of vRNP export.

### Crm1 is relocalized to chromatin-bound sites of vRNP export complexes

**Figure 5:**
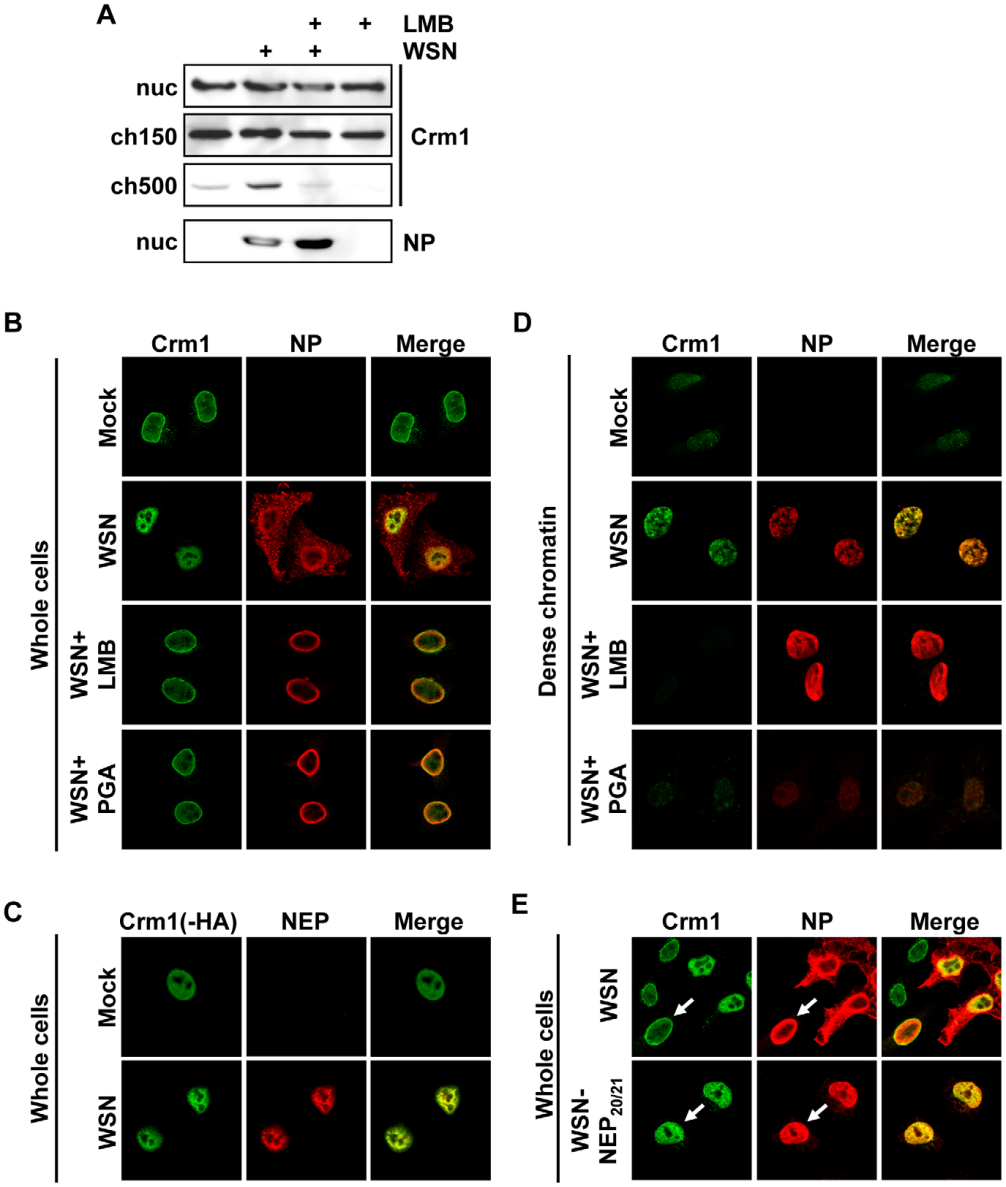
Relocalization of Crm1 to dense chromatin after influenza virus infection. (A) 1×10^9^ HeLa cells were infected with WSN at an MOI of 3 or mock-infected. At 3hpi, cells were treated with LMB or EtOH, and at 9hpi cells were fractionated. Lysates from the ch500 fraction were analyzed by Western blot for Crm1 or NP. (B) HeLa cells grown on coverslips were infected with WSN at an MOI of 3 or mock-infected. Cells were treated with EtOH, LMB or PGA at 3hpi, fixed at 9hpi, and analyzed by immunofluorescence using antibodies against NP and Crm1. (C) HeLa cells were transfected with Crm1-HA, infected with WSN at an MOI of 3 or mock-infected for 9 h, and analyzed by IFA using antibodies against NEP or HA. (D) Cells were infected and treated as in (B), then fractionated to reveal dense chromatin before fixation and immunofluorescence. (E) HeLa cells were infected with WSN or WSN-NEP_20/21_ for 9 h, and analyzed by IFA using antibodies against NP or Crm1.

Our data suggested that tight association of influenza vRNPs with chromatin is a prerequisite for their subsequent export, which has not been described for exported cellular proteins. To test whether vRNP chromatin association affects the host cell export machinery, we analyzed the distribution of Crm1 in the nucleus of infected cells. Biochemical fractionation of virus-infected cells at 9 hpi revealed an accumulation of Crm1 in the ch500 fraction, which could be reversed upon LMB treatment (Fig. 5A). Similarly, IFA showed a pronounced relocalization of Crm1 from the nuclear periphery to the interior of the nucleus, which was reversible after LMB or PGA treatment (Fig. 5B). This relocalization was also observed in infected A549 and MDCK cells (data not shown). Although Crm1 co-localized only partially with influenza NP in whole cells (Fig. 5B, middle panels), there was significant co-localization of Crm1-HA with NEP (Fig. 5C). This relocalization of Crm1 upon viral infection correlated with an increase of Crm1 at the sites of NP distribution on the dense chromatin (Fig. 5D), which was also reversed upon LMB or PGA treatment. Interestingly, in WSN-wt-infected cells, redistribution of Crm1 was only observed in cells containing cytoplasmic NP, implying that Crm1 relocalization and active vRNP export are tightly linked (Fig. 5E, upper panels). In contrast, we observed many WSN-NEP_20/21_-infected cells displaying Crm1 relocalization without cytoplasmic NP staining (Fig. 5E, lower panels), suggesting that Crm1 relocalization is not strictly dependent on robust vRNP export. Thus, Crm1 relocalizes to the sites on dense chromatin where vRNP export complexes also accumulate.

### Export of a non-viral Crm1 substrate is impaired in influenza virus-infected cells

**Figure 6:**
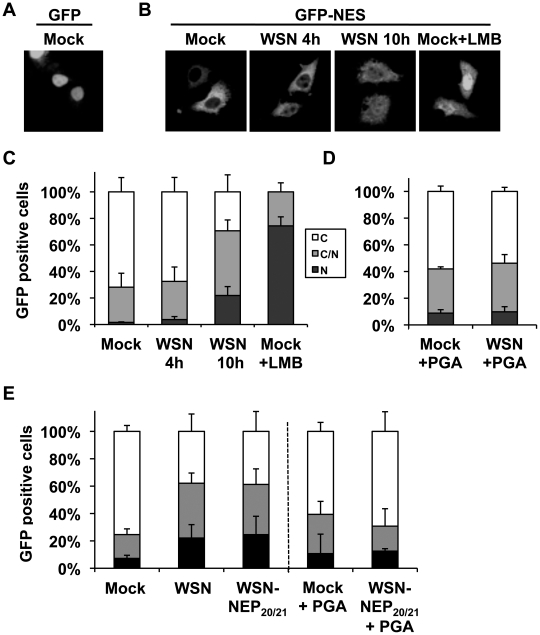
Influenza infection impairs nuclear export of a Crm1-dependent protein. (A) HeLa cells grown on coverslips were transfected with GFP, mock infected, and fixed. (B) HeLa cells grown on coverslips were transfected with GFP-NES and either infected with WSN at an MOI of 3, mock-infected, or treated with LMB. At the times indicated, cells were fixed, and GFP was detected by confocal microscopy. (C) Cells from (B) were scored for the percentage of GFP-expressing cells which displayed either predominantly cytoplasmic (C), approximately equal cytoplasmic and nuclear (C/N), or predominantly nuclear (N) GFP distribution. Values are the average of three independent assays. (D) HeLa cells grown on coverslips were transfected with GFP-NES and either infected or mock-infected at an MOI of 3 for 10 h, and treated with PGA at 3hpi. At the times indicated, cells were fixed, and GFP localization was scored as in (C). (E) HeLa cells transfected with GFP-NES were infected with WSN or WSN-NEP_20/21_ at an MOI of 3 for 9 h, and some treated with PGA at 3hpi. GFP localization was scored as in (C).

Based on the ability of influenza vRNPs to sequester at least a portion of cellular Crm1 on the chromatin, we postulated that the resulting depletion of free Crm1 might impair the export of cellular Crm1 substrates. To test this hypothesis, we measured the export efficiency of a non-viral Crm1 substrate in influenza virus-infected cells compared to uninfected cells. Due to the targeting of many cellular Crm1 substrates to the nucleus during influenza virus infection, we used GFP containing an HIV Rev-derived NES (GFP-NES) as a marker for Crm1-mediated export. Low-level GFP expression in HeLa cells led to a predominantly nuclear localization, similar to previous findings [32] (Fig. 6A). In contrast, GFP-NES localized almost exclusively in the cytoplasm (Fig. 6B, left panel), and thus provided a suitable measure of an actively-exported Crm1 substrate, as has been previously reported [33]. Upon influenza virus infection, retention of GFP-NES in the nucleus was observed after 10 h of infection (during vRNP export) but not 4 h (Fig. 6B + C), which was also observed in A549 cells (data not shown). Interestingly, while the majority of virus-infected cells contained GFP-NES both in the cytoplasm and the nucleus, treatment of GFP-NES-transfected cells with LMB led to a predominantly nuclear accumulation (Fig. 6B+C). These results suggest that influenza virus infection can impair, but not shut off, the export of non-viral Crm1 substrates. To test whether this impairment of cellular export was dependent on chromatin accumulation of influenza vRNPs, we treated infected cells with PGA, which blocks chromatin association but not total nuclear accumulation of vRNPs (Fig. 4A). Although some GFP-NES was retained in mock-infected cells upon PGA treatment (Fig. 6D), no difference in nuclear retention was observed between mock-infected and influenza virus-infected cells. Thus, impairment of cellular export by influenza virus is not only dependent on accumulation of nuclear vRNPs, but also on their association with the ch500 fraction.

As shown in Fig. 5E, infection with the mutant WSN-NEP_20/21_ could induce Crm1 relocalization to an equivalent extent as wt WSN, despite reduced vRNP export. In GFP-NES transfected cells, WSN-NEP_20/21_ inhibited GFP-NES nuclear export equally as WSN (Fig. 6E). Treatment of these cells with PGA similarly abrogated any impairment in GFP-NES export (Fig. 6E, right side). Thus, impairment of cellular Crm1-dependent export by influenza virus requires vRNP association with chromatin, but not robust vRNP export.

### vRNPs associate with Rcc1 on chromatin

**Figure 7:**
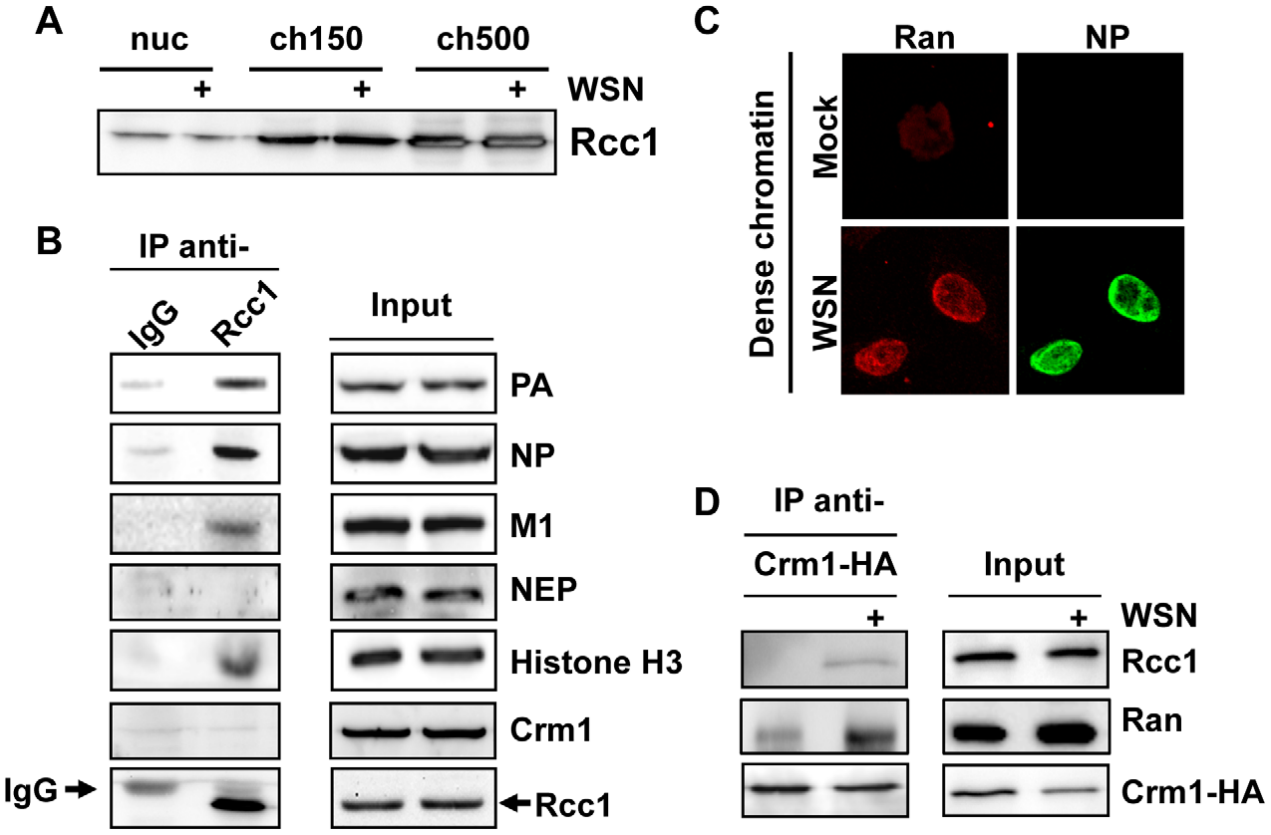
Association of Rcc1 with vRNPs and Crm1. (A) 1×10^9^ HeLa cells were infected with WSN at an MOI of 3 or mock-infected for 9 h. Cells were fractionated, and nuclear lysates were analyzed for distribution of Rcc1. (B) 1×10^9^ HeLa cells were infected with WSN at an MOI of 3 for 9 h. Nuclease-digested lysates were incubated with anti-Rcc1 antibodies or pre-immune serum (IgG) and precipitated with Protein G agarose. Immunoprecipitates and input lysates were analyzed by Western blot for the indicated proteins. (C) HeLa cells grown on coverslips were infected with WSN at an MOI of 3 or mock-infected. Whole cells were fixed at 9hpi, and analyzed by IFA using antibodies against NP and Ran. (D) 2×10^8^ HeLa cells were transfected with an expression plasmid encoding HA-tagged Crm1. 16 h post-transfection, cells were infected with WSN at an MOI of 3 or mock-infected for 9 h. Cells were fractionated, and chromatin lysates were immunoprecipitated using monoclonal HA antibodies. Lysates and precipitates were normalized for Crm1 amounts (to accurately judge amounts of co-precipitated Ran and Rcc1) and analyzed by Western blot for HA-tagged Crm1 or endogenous Rcc1.

Crm1-dependent nuclear export is driven by association of Crm1 with the small GTPase Ran in its GTP-bound state. Exchange of RanGDP for RanGTP is a chromatin-bound process [11] which is catalyzed by the guanine exchange factor Rcc1, during which a putative Crm1-Ran-Rcc1 complex can transiently form [12]. We were therefore interested whether chromatin-targeting of influenza vRNP export complexes might bring them into proximity with the sites of RanGTP regeneration. First, we performed a biochemical fractionation of uninfected or influenza virus-infected cells to determine the distribution of Rcc1 on chromatin in our fractionation protocol. Interestingly, the highest Rcc1 concentration was found in chromatin extracted with 500 mM NaCl (ch500, Fig. 7A), corresponding to the fraction containing vRNP export complexes. Next, we tested whether Rcc1 and vRNP export complexes can associate on chromatin. Immunoprecipitation of chromatin-extracted, infected cells using Rcc1-specific antibodies co-purified NP, PA, and M1, as well as histone H3 (Fig. 7B). Neither NEP nor Crm1 could be detected in the immunopurified complexes (data not shown). In summary, Rcc1 and components of vRNP export complexes can form a complex, although it is unclear whether this interaction is direct, or indirect via nucleosomes.

Based on our finding that Crm1 can be retained on chromatin during influenza virus infection (Fig. 5D), we tested whether infection causes an increase in formation or stability of Crm1-Ran-Rcc1 complexes on chromatin. Virus infection indeed led to accumulation of Ran on dense chromatin (Fig. 7C), partially colocalizing with NP. We then immunoprecipitated HA-tagged Crm1 from uninfected or infected cells after chromatin extraction and measured the amount of bound Ran or Rcc1 by Western blot. At 9 hpi, more Ran and Rcc1 was co-immunoprecipitated by Crm1-HA than in uninfected cells (Fig. 7D). Corresponding to our previous observations, no viral proteins could be detected in the Crm1-HA-bound complexes (data not shown).

## Discussion

Unlike most negative-strand RNA viruses, influenza viruses require a functional nuclear export system for nucleocytoplasmic transport of its vRNPs for their incorporation into progeny virions at late time points of infection. Here, we demonstrate that exported vRNPs are assembled on a chromatin fraction which can only be extracted at high salt concentration. Inhibition of Crm1-mediated nuclear export leads to an accumulation of vRNPs containing M1 and NEP on this chromatin fraction, while sequestration of M1 and vRNPs by Hsp70 overexpression prevents their association with this chromatin fraction, thus leading to a lack of vRNP export. Accumulation of vRNPs on the chromatin immediately prior to their export leads to retention of the cellular export proteins Crm1 and Ran at these chromatin-bound sites of vRNP export complex assembly. This relocalization of Crm1 results in a decrease in the nuclear export of other Crm1 substrates. Intriguingly, these sites of vRNP export complex assembly are physically associated with the Ran nucleotide exchange factor Rcc1, and influenza virus infection leads to an increase in the association between Rcc1, Ran, and Crm1.

Several lines of evidence confirm that the vRNPs purified from 500 mM NaCl-extractable chromatin are genuine vRNP export complex precursors. First, these complexes contain both M1 and NEP (Fig. 1D). Although the amount of co-purified NEP was relatively low, it is unclear whether a large amount of the protein is required for export [5]; conversely, vRNPs from the ch500 fraction contained the highest concentration of M1, which is a major driver of vRNP nuclear export [1], [4]. Second, only these complexes are enriched after vRNP nuclear export is blocked either through inhibition of cellular export (Fig. 1D) or mutation of the NES required for vRNP export (WSN-NEP_20/21_, Fig. 3A + B). Third, while IFA of unfractionated cells revealed a large discrepancy between the distributions of PA and NP in the nucleus (Fig. 2B), extracting all proteins except those bound to chromatin with high affinity showed a colocalization between PA, NP, M1, and NEP, all involved in vRNP export (Figs. 2B + C and data not shown). The exposure of previously-hidden epitopes through extraction has been previously described [29], and likely explains the differences in NP distribution observed between unextracted and chromatin-extracted cells on coverslips. Confirming the biochemical fractionation results, the accumulation of PA, NP, and NEP on dense chromatin also increased after LMB treatment, while no difference was observed on total chromatin or in unextracted cells (Fig. 2B + C). Thus, the vRNP export complex appears to assemble on dense chromatin, from which it translocates to the cytoplasm in a process most likely too transient to capture using our steady-state methods.

One open question in our data is where in the nucleus vRNP export complexes are synthesized. Our finding that PGA treatment reduces the association of vRNPs with chromatin (Fig. 4) suggests that vRNPs are synthesized elsewhere in the nucleus and subsequently associate with the ch500 fraction after synthesis in an M1-dependent manner. Interestingly, previous studies using in vitro transcription assays demonstrated that some portion of vRNA can be synthesized on “nuclear matrix”-like, high-salt insensitive chromatin [18], [34], suggesting the possibility that both vRNP synthesis and export complex assembly are chromatin-bound.

Interestingly, we also purified vRNPs containing NEP from the nucleoplasm (Figs. 1D, 4A). However, these complexes contained no detectable M1, and were not enriched after LMB treatment. Recently, several reports have described a role for NEP in viral RNA replication, including evidence that NEP by itself can increase cRNA and vRNA synthesis in a vRNP reconstitution assay [35], [36]. We therefore speculate that these NEP-containing vRNPs are not exported from the nucleus, but may be involved in the synthesis of full-length viral genomic RNA or cRNA.

Both Crm1 and Ran are very dynamic proteins, which constantly “scan” the nucleus for potential export substrates via diffusion through the soluble region of the nucleus [37], [38]. Although little is known about the nuclear locations at which Crm1 associates with its cargo, many Crm1 substrates, such as nuclear receptors, are very mobile proteins which associate transiently with actively transcribing chromatin [39]. In contrast, RanGTP regeneration by Rcc1 has been shown to be a tightly chromatin-bound process [38], [40]. Our fractionation data suggest that Rcc1 associates with a dense, less transcriptionally-active chromatin fraction, raising the possibility that RanGTP regeneration and Crm1 cargo recognition occur at biochemically and spatially distinct domains in uninfected cells. This separation would provide an unexploited “niche” for influenza vRNPs to access Crm1-RanGTP complexes before they diffuse to other sites of the nucleus. While we cannot determine whether there is a direct contact between Rcc1 and vRNPs (Fig. 7B), both complexes appear to be located on the same nucleosomes, and the ability of influenza virus infection to stabilize the Rcc1-Ran-Crm1 complex (Fig. 7D) suggests that chromatin docking of vRNP export complexes may directly elicit an alteration of Rcc1. The lack of co-precipitated Crm1 or NEP with Rcc1-vRNP complexes (Fig. 7B) is likely due to the very transient nature of both Crm1 and NEP interactions (unpublished observations). Moreover, there is a possibility that different transient complexes between vRNPs, Crm1, and Rcc1 are assembled sequentially on nucleosomes, which could contribute to the asymmetry between Crm1 and Rcc1 immunoprecipitation data.

**Figure 8:**
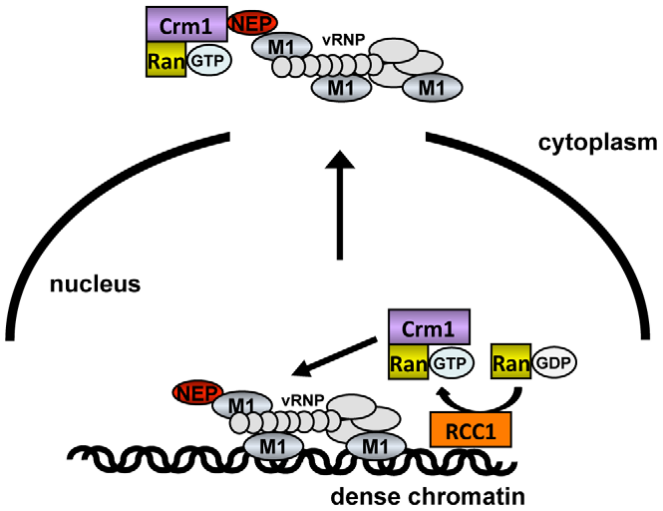
Model of vRNP export. vRNPs (light gray) complexed with M1 (blue) and NEP (red) associate with dense chromatin in proximity to Rcc1 (orange). During or immediately after generation of RanGTP (purple), the proximity of vRNPs to Rcc1 may allow vRNPs access to a RanGTP-Crm1 complex before it can diffuse to other sites in the nucleus.

Based on our findings, we propose that influenza vRNPs gain preferential access to newly-regenerated host cell export machinery through specific chromatin targeting, which confers a physical proximity to the sites of Ran nucleotide exchange (see model in Fig. 8). During late time points of infection, vRNPs associate transiently with dense chromatin, and assemble with M1 and NEP at these sites (Fig. 1D, Fig. 2B+C). This targeting is likely mediated by M1 itself, since M1 can bind to chromatin with high affinity [19], [41], and is sequestered from chromatin after Hsp70 overexpression (Fig. 4B). These chromatin-bound sites of vRNP export complex assembly are associated either directly or via nucleosomes with Rcc1, on which a transient Crm1-Ran-Rcc1 complex is formed in order to regenerate RanGTP from RanGDP [12]. We postulate that the proximity of these components allows influenza vRNPs to “snatch” newly-generated Crm1-RanGTP complexes before they can diffuse to cellular NES-containing substrates at distal nuclear sites (Fig. 8).

This sequestering of Crm1/RanGTP on the chromatin is a likely cause of GFP-NES retention in the nucleus of infected cells (Fig. 6). Although GFP-NES is not a cellular Crm1 substrate, it is generally accepted as a marker for Crm1 activity [42], and was expressed at low levels in our assay. By retaining a portion of Crm1 on the chromatin, influenza virus infection may reduce the pool of free, soluble Crm1 available to facilitate the export of other proteins more distant from Rcc1. Unlike other RNA viruses [43], [44], influenza virus infection impaired but did not completely block nuclear export, thus keeping intact a cellular system required for viral propagation. Impairment of cellular export seems to be a specific consequence of vRNP association with chromatin: accumulation of vRNPs in the nucleus without chromatin association did not affect export (Fig. 6C, PGA), while vRNP association with chromatin in the absence of vRNP nuclear export led to a decrease in cellular export (Fig. 6D, WSN-NEP_20/21_). Based on these findings, we postulate that influenza vRNPs gain preferential access to Crm1 in the nucleus, to the detriment of cellular export substrates.

In summary, we show here that influenza viruses use a unique chromatin-targeting strategy to facilitate vRNP export. The chromatin association of vRNPs leads to a retention of Crm1 and Ran, which in turn impairs the ability of other Crm1 substrates to be exported. Thus, we postulate that influenza vRNPs gain an advantage in nuclear export over cellular substrates through chromatin association and subsequent Crm1 “snatching.” While other examples of host-cell hijacking by viruses involve higher binding affinities or shutting off of cellular processes, influenza viruses appear to exploit an unused locational niche in gaining preferential access to the cellular export machinery.

## Materials and Methods

### Ethics statement

All animal experiments were performed in compliance with the German guidelines according to § 8a Animal Welfare Act of 18. May 2006 (BGBI.I, p. 1206), European Union guidelines 86/609/EWG of 24.11.1986 and according to the European Agreement of 18.3.1996 for protection of animal trials and other for scientific purposes used vertebrates of 11.12.1990 (BGBI. II S. 1486) and NIH/OLAW Animal Welfare Assurance #A5755-01″. The animal welfare committees of the Robert Koch Institute and the Institute of Biotechnology of Vilnius University approved all animal experiments.

### Antibodies

Hybridomas producing monoclonal antibodies (MAbs) against PA (A/WSN/33) were generated essentially as described by ([45]). Eight-week-old female BALB/c mice (obtained from a breeding colony at the Institute for Innovative Medicine, Vilnius, Lithuania) were immunized at days 0, 28 and 56 by a subcutaneous injection of 50 µg of recombinant purified PA [46]. The specificity of MAbs was tested by an indirect ELISA. Twenty five hybridomas giving initial ELISA reactivities (OD450) against full-length recombinant PA OD450 >2.0 were identified and subcloned. After subcloning, 22 stable hybridoma cell lines producing MAbs of IgG isotype IgG1 subtype were obtained. The MAb produced by clone 1E7, used in this study, showed high reactive against recombinant full-length C9 terminally hexahistidine-tagged Flu A PA both by ELISA and Western blot (data not shown). The MAb 1E7 did not cross-react using purified C-terminally His-tagged Flu B PA and lysates of either non-transformed yeast cells.

Influenza-specific anti-NP antibodies have been previously described [47], while polyclonal anti-NEP antibody was generated by immunizing a rabbit with recombinant glutathione-S-transferase fused to NEP derived from the influenza A/PR/8/34 virus.

All other antibodies were purchased: anti-M1 (ab22396), anti-histone H3 (ab1791), anti-HMGB1 (ab18256), and anti-Rcc1 (ab54600) from Abcam; anti-Crm1 (sc-5595) from Santa Cruz Biotechnology; anti-TFIIB from Euromedex; anti-Pol II (8WG16) from Covance; anti-tubulin from Sigma-Aldrich; and anti-Ran (610341) from BD Transduction Laboratories. All secondary antibodies were purchased from Jackson Immunoresearch.

### Cell lines and chemicals

HeLa, A549 and MDCK cells were cultured in DMEM containing penicillin and streptomycin.

Prostaglandin A (PGA) was purchased from Sigma and stored at a stock concentration of 5 mg/ml in ethanol. Leptomycin B was purchased from Enzo Biochemicals and stored at a stock concentration of 5 µg/ml in ethanol.

### Virus strains and infections

Recombinant viruses rWSN-PB2-Strep and rWSN (untagged) were generated as previously described [24]. Viruses were diluted in phosphate-buffered saline (PBS) containing 0.3% bovine serum albumin (BSA), and cells were infected for 1 h at 37°C. Virus titers from supernatants were performed on MDCK cells using standard plaque assay protocol. WSN-NEP_20/21_ was generated by site-directed mutagenesis based on the sequence in ([30]) in the plasmid pPOLI-NS [48].

### Plasmids

Plasmid pCA-GFP-NES was generated from pCA-Flag-GFP [46] using a reverse primer containing the HIV Rev NES sequence (LQLPPLERLTLD) preceded by a linker ecoding three glycine residues and followed by a stop codon and a BglII restriction site. PCR was performed using this primer and a GFP-specific forward primer, and the product was digested and ligated into the digested vector.

### Subcellular fractionation

10^9^ HeLa cells were washed and pelleted in PBS. The cell pellet was resuspended in 10 ml sucrose buffer (10 mM HEPES pH 7.9, 10 mM KCl, 2 mM Mg acetate, 3 mM CaCl2, 340 mM sucrose, 1 mM DTT, 1 mM PMSF), and cells were incubated 10 min. on ice. NP-40 was then added to a final concentration of 0.5%, followed by vortexing for 15 sec and centrifugation for 10 min at 4°C at 3,500 *g*. Supernatant was saved as the cytoplasmic fraction, and the nuclear pellet was washed once with 5 ml sucrose buffer. The nuclear pellet was then resuspended in 1.5 ml nucleoplasm extraction buffer (50 mM HEPES pH 7.9, 150 mM potassium acetate, 1.5 mM MgCl2, 0.1% NP-40, 1 mM DTT, 1 mM PMSF), transferred to an all-glass 4 ml Dounce homogenizer with a tight-fitting pestle, and homogenized with 20 strokes. Homogenate was then incubated 20 min at 4°C on a rotating wheel, followed by centrifugation for 10 min at 4°C at 14,000 rpm in a microcentrifuge. The supernatant was saved as the nucleoplasmic fraction, and the pellet was resuspended in 1.5 ml nuclease incubation buffer (50 mM HEPES pH 7.9, 10 mM NaCl, 1.5 mM MgCl_2_, 1 mM DTT, 1 mM PMSF) containing 100 U/ml Benzonase nuclease (Novagen). Chromatin was digested for 10′ at 37°C, followed by addition of NaCl to 150 mM and further incubation 20 min on ice. Digested lysate was then centrifuged 10 min at 4°C at 14,000 rpm, and supernatant was saved as the low-salt chromatin fraction. The pellet was resuspended in 1.5 ml chromatin extraction buffer (50 mM HEPES pH 7.9, 500 mM NaCl, 1.5 mM MgCl_2_, 0.1% Triton X-100, 1 mM DTT, 1 mM PMSF) and incubated 20 min on ice, followed by centrifugation as above. The supernatant was saved as the high-salt chromatin fraction.

### Strep-tag purification

100 µl Strep-Tactin Sepharose (IBA) was washed with 30 volumes strep wash buffer (20 mM HEPES pH 7.9, 150 mM NaCl, 1 mM EDTA) and added to fractionated lysates. Lysates were incubated with beads for 1 h at 4°C on a rotating wheel. Beads were collected by centrifugation, washed three times with strep wash buffer (containing 500 mM NaCl for high-salt chromatin samples), and eluted with 100 µl strep wash buffer containing 2.5 mM desthiobiotin (IBA). Eluates were diluted 1∶1 with 2x Lämmli buffer for further analysis.

### Primer extension

For primer extension, cells were first fractionated as above, except that Benzonase nuclease was replaced with 20 U/ml RNase-free DNase I (Fermentas). RNA was then extracted from each fraction using a Nucleospin RNA II Kit (Macherey-Nagel). Primer extension was performed using WSN NA-specific primers as previously described [35].

### In situ fractionation and immunofluorescence

HeLa cells grown on coverslips were washed once with cold PBS, then incubated with 250 µl/well CSK (50 mM PIPES pH 6.8, 100 mM NaCl, 300 mM sucrose, 3 mM MgCl_2_, 1 mM PMSF) containing 0.5% Triton X-100 for 3 min. on ice, followed by a brief wash with CSK. Cells were then incubated with 250 µl DB (50 mM PIPES pH 6.8, 50 mM NaCl, 300 mM sucrose, 3 mM MgCl_2_, 1 mM CaCl_2_, 1 mM PMSF) containing 20 U/ml RNase-free DNase I (Fermentas) for 10 min. at 37°C. This buffer was removed, and cells were incubated in DB containing 250 mM NaCl for 3 min. on ice. Experiments were performed in triplicate, and one coverslip each was fixed before the extraction, after detergent extraction, and after chromatin extraction. Cells were fixed 10′ with 3% paraformaldehyde, followed by standard immunofluorescence. DNA was stained using ToPro3 (Invitrogen). For GFP-NES experiments, HeLa cells were on coverslips in 24-well plates were transfected with 200 ng/well pCA-GFP-NES. After infection, coverslips were mounted on slides directly after fixation. Optical sections were obtained using a Leica TCS confocal microscope.

### Immunoprecipitations

HeLa cells were transfected with a plasmid encoding HA-tagged Crm1 ([49], kindly provided by R. Kehlenbach, Göttingen, Germany) using Nanofectin (PAA). 24 h after transfection, cells were infected for 9 h. Fractionation was then performed as above, except that high-salt chromatin extraction was performed using 300 mM NaCl instead of 500 mM to prevent disruption of Crm1 complexes. High salt chromatin lysates were incubated with anti-HA agarose (Sigma-Aldrich) for 1 h at 4°C on a rotating wheel, then washed three times with strep wash buffer containing 300 mM NaCl. Bound complexes were eluted using Lämmli buffer and analyzed by Western blot.

For anti-Rcc1 immunoprecipitation, cells were infected 9 h, then lysed with lysis buffer (50 mM Tris pH 7.5, 250 mM NaCl, 2 mM MgCl2, 0.5% NP-40, 1 mM PMSF, 1 mM DTT) containing 100 U Benzonase per ml 20 min. on ice. Lysates were incubated with anti-Rcc1 (Santa Cruz) and precipitated using Protein G agarose (Pierce). Beads were washed 3 times with lysis buffer without NP-40 and eluted with Lämmli buffer.

## Supporting Information

Figure S1 **Strep-purification of vRNPS.** (A) A549 cells were infected with rWSN or rWSN-PB2-Strep at an MOI of 0.001, and infectious supernatant titer was determined by plaque assay at times shown. Error bars represent standard deviation from 3 independent experiments. Infectious titers were not measurable from HeLa cells, but equal infection kinetics in these cells was confirmed using immunofluorescence and Western Blot analyses (not shown). (B) HeLa cells were infected with rWSN or rWSN-PB2-Strep at an MOI of 3. At 6 hpi, cells were lysed and Strep purification was performed as described in Materials and Methods. Lysates and eluates were analyzed by Western blot for indicated proteins. Equal amounts of lysate from before Strep purification (left lanes, “Lysate”) and after purification (middle lanes, “Unbound”) were loaded. (C) Eluates from (B) were visualized by silver staining. Upper bands marked by asterisk were confirmed as RNA by RNase digestion (not shown). (D) 1×10^9^ HeLa cells were infected with rWSN or rWSN-PB2-Strep at an MOI of 3 for 9 h before subcellular fractionation. Equal amounts of protein from the rWSN-PB2-Strep-infected samples were analyzed by Western blot for distribution of PA or NP. (E) Eluates from Strep purification using the lysates from (D) were analyzed by SDS-PAGE and staining with silver. (F) Eluates from (E) were analyzed by Western blot for PA or NP amounts, which were quantified by densitometry, and the ratio of NP:PA in each fraction was calculated.
(TIF)

Figure S2 **Colocalization of NP and histone H2B.** HeLa cells on coverslips were transfected with a plasmid expressing H2B-GFP, and 16 h later infected with WSN at an MOI of 3 for 9 h. Whole cells (upper panels) or fractionated cells (lower panels, dense chromatin) were analyzed by IFA for NP and H2B localization.
(TIF)

Figure S3 **Protein accumulation after LMB or PGA treatment.** HeLa cells were infected with WSN at an MOI of 3 for 7 h, and treated with EtOH, LMB, or PGA at 3hpi. Whole-cell lysates were analyzed by Western blot for accumulation of PA or NP.
(TIF)
