# Supplementary figures and images for: Influenza Virus Ribonucleoprotein Complexes Gain Preferential Access to Cellular Export Machinery through Chromatin Targeting
Source: PLoS Pathog. 2011 Sep 1;7(9):e1002187. doi: 10.1371/journal.ppat.1002187 (PMC3164630; doi:10.1371/journal.ppat.1002187)

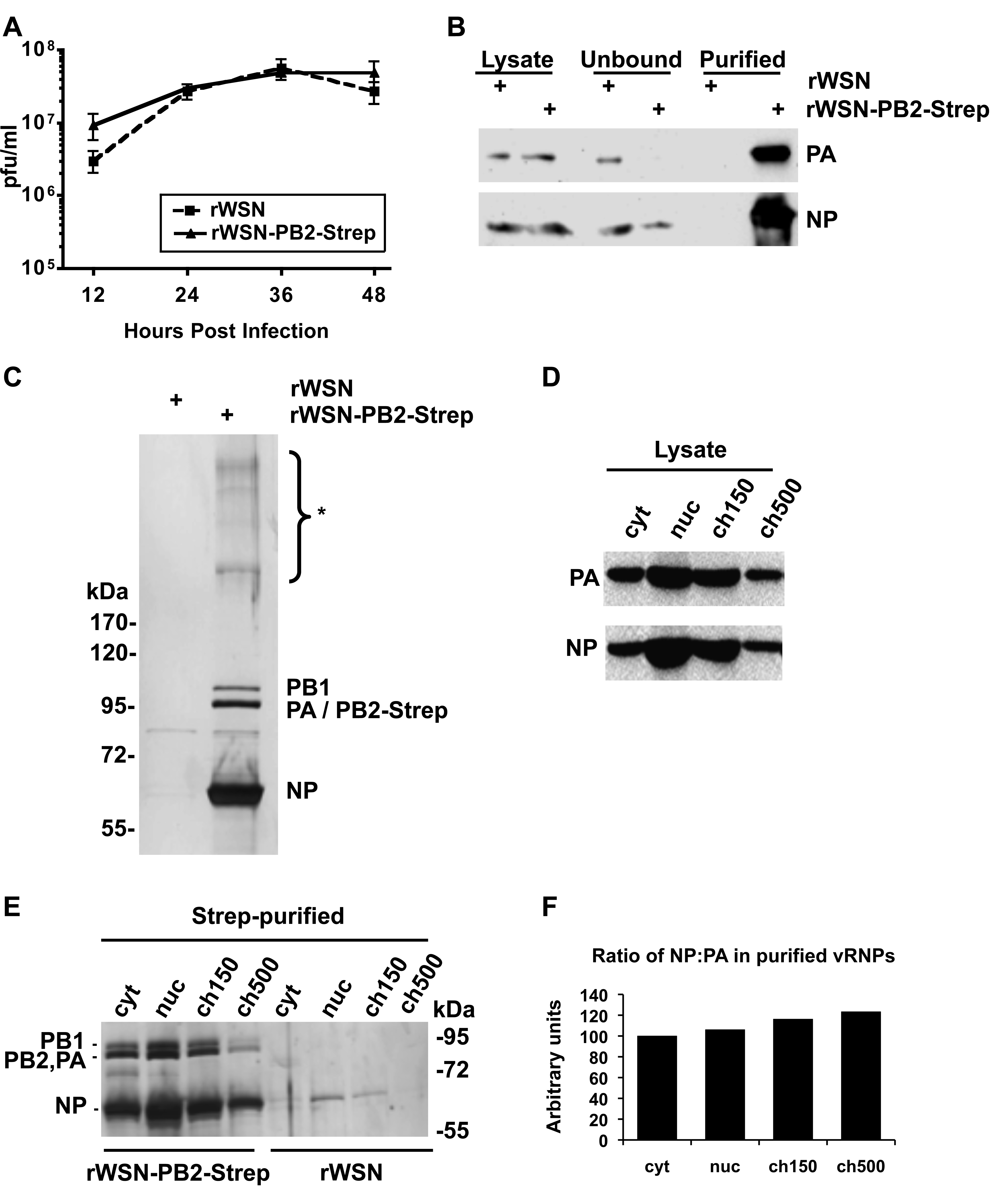

Supplement: Figure S1 — Strep-purification of vRNPS. (A) A549 cells were infected with rWSN or rWSN-PB2-Strep at an MOI of 0.001, and infectious supernatant titer was determined by plaque assay at times shown. Error bars represent standard deviation from 3 independent experiments. Infectious titers were not measurable from HeLa cells, but equal infection kinetics in these cells was confirmed using immunofluorescence and Western Blot analyses (not shown). (B) HeLa cells were infected with rWSN or rWSN-PB2-Strep at an MOI of 3. At 6 hpi, cells were lysed and Strep purification was performed as described in Materials and Methods. Lysates and eluates were analyzed by Western blot for indicated proteins. Equal amounts of lysate from before Strep purification (left lanes, “Lysate”) and after purification (middle lanes, “Unbound”) were loaded. (C) Eluates from (B) were visualized by silver staining. Upper bands marked by asterisk were confirmed as RNA by RNase digestion (not shown). (D) 1×10^9^ HeLa cells were infected with rWSN or rWSN-PB2-Strep at an MOI of 3 for 9 h before subcellular fractionation. Equal amounts of protein from the rWSN-PB2-Strep-infected samples were analyzed by Western blot for distribution of PA or NP. (E) Eluates from Strep purification using the lysates from (D) were analyzed by SDS-PAGE and staining with silver. (F) Eluates from (E) were analyzed by Western blot for PA or NP amounts, which were quantified by densitometry, and the ratio of NP:PA in each fraction was calculated. (TIF) [file ppat.1002187.s001.tif]

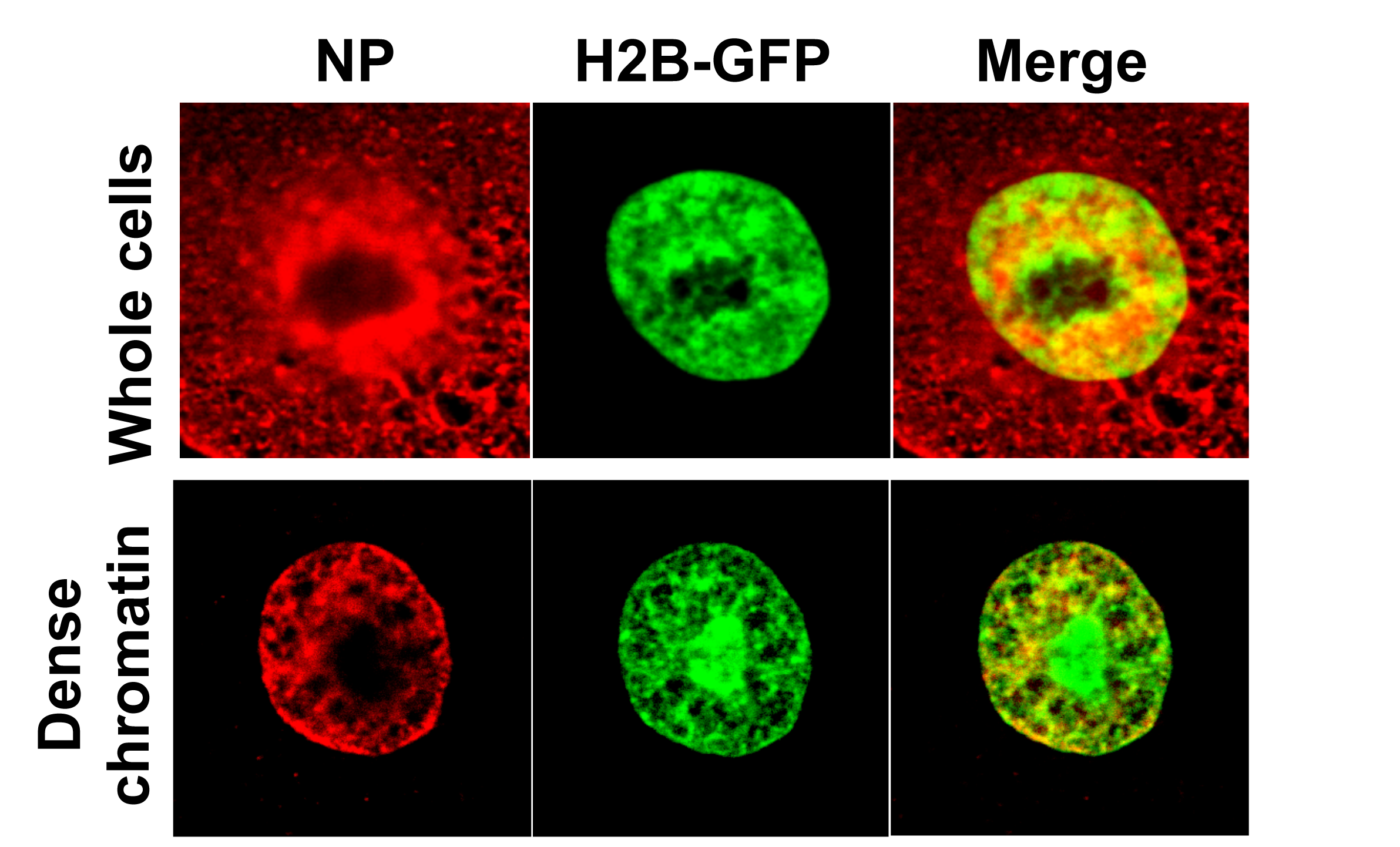

Supplement: Figure S2 — Colocalization of NP and histone H2B. HeLa cells on coverslips were transfected with a plasmid expressing H2B-GFP, and 16 h later infected with WSN at an MOI of 3 for 9 h. Whole cells (upper panels) or fractionated cells (lower panels, dense chromatin) were analyzed by IFA for NP and H2B localization. (TIF) [file ppat.1002187.s002.tif]

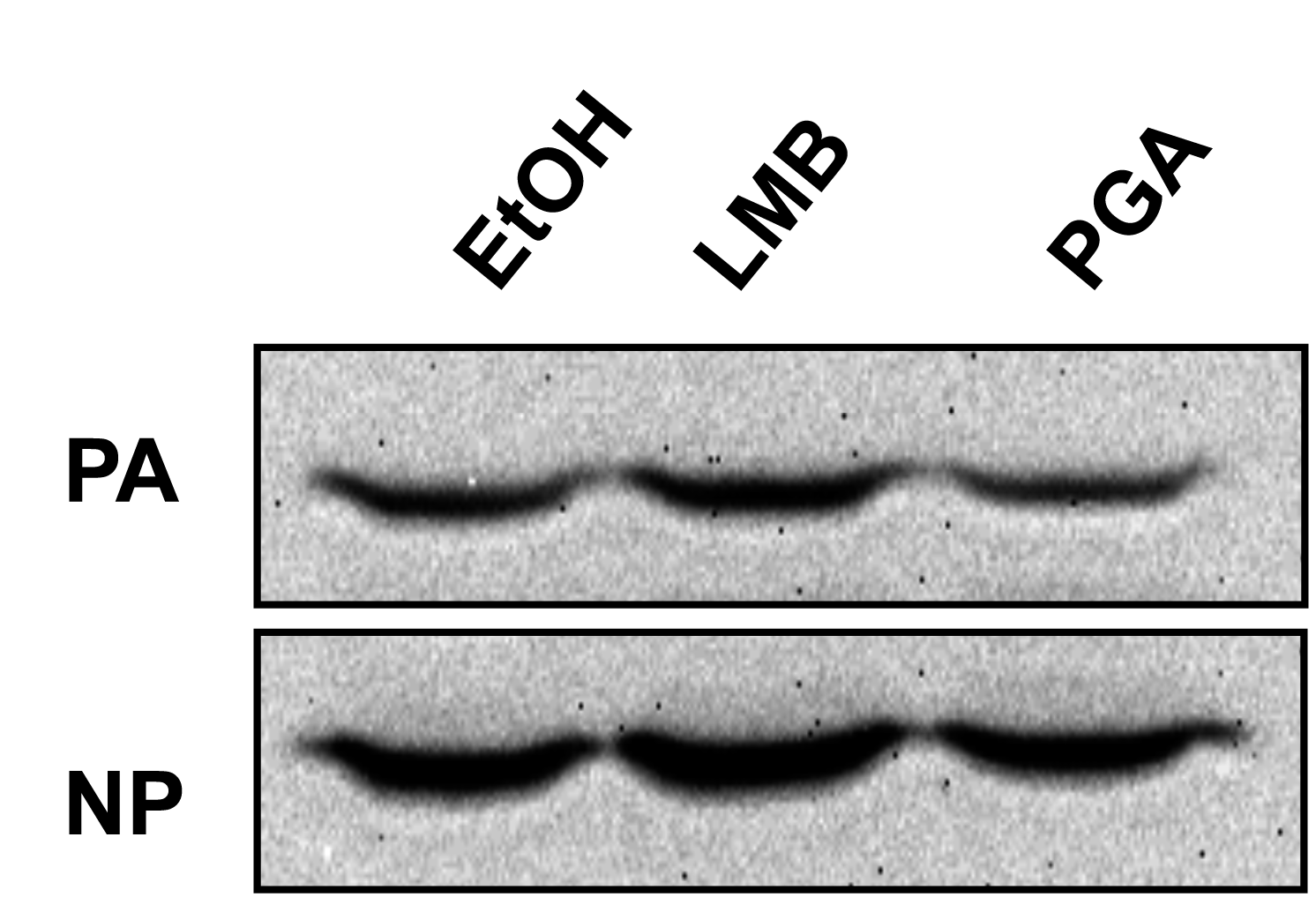

Supplement: Figure S3 — Protein accumulation after LMB or PGA treatment. HeLa cells were infected with WSN at an MOI of 3 for 7 h, and treated with EtOH, LMB, or PGA at 3hpi. Whole-cell lysates were analyzed by Western blot for accumulation of PA or NP. (TIF) [file ppat.1002187.s003.tif]
